# *in vitro* Decellularization of Rabbit Lung Tissue

**Published:** 2013-05-05

**Authors:** Nasser Mahdavi Shahri, Javad Baharara, Mahan Takbiri, Saeedeh Khajeh Ahmadi

**Affiliations:** 1 Department of Biology, Mashhad Branch, Islamic Azad University, Mashhad, Iran; 2 Oral and Maxillofacial Diseases Research Center, Faculty of Dentistry, Mashhad University of Medical Sciences, Mashhad, Iran

**Keywords:** Tissue Engineering, Lung Organ, Scaffold

## Abstract

**Objective:**

The bioscaffold can be used in tissue engineering and regenerative medicine. The scaffolds used in tissue engineering must have high porosity to facilitate accelerated angiogenesis for feeding cells and repelling cell waste outside the scaffold. In this experimental study, we attempted to produce lung three-dimensional scaffold and assay its effect on cell penetration and migration.

**Materials and Methods:**

In an experimental study, rabbit lung tissue was decellularized and used as a scaffold for rabbit blastema cells. The scaffolds were studied on the 15^th^ day after culturing.

**Results:**

Microscopic features revealed high porosity in the lung tissue scaffold. Electron microscopic imaging also showed collagen and elastin were intact, which are important properties in scaffolds designed for tissue engineering. Migration and permeation of blastema cells into the lung tissue scaffold was also observed.

**Conclusion:**

Rabbit lung tissue scaffolds have high porosity. Blastema cells successfully migrated toward and permeated the scaffold inside.

## Introduction

Tissue engineering is a new field of research, which combines disciplines such as cell biology, biochemistry, molecular biology, chemical engineering, and bioengineering in the reconstruction of tissues (1, 2).

In the recent years, tissue engineering has made rapid progress to the extent that it is increasingly seen as the biological successor to organs for the reconstruction of tissue damage. Tissue engineering differs from common standard treatment methods because specifically designed tissues are transplanted to the patient (3). It is now recognized as a new field with special aims (4). Factors required for the success of tissue engineering are a suitable scaffold and a reliable source of cells.

The main cellular source to date has been stem cells, but, blastema cells, which are undifferentiated cells having embryonic cell specifications, can also provide a source of cells for tissue engineering (5).

A study in 2008 showed that blastema cells derived from cell dedifferentiation at the site of wounds can be differentiated into many kinds of cells (6). Recently, a tissue engineering study of the potential of using stem cells in a scaffoldled to a newer method of lung tissue transplantation. Animal models and clinical study suggest the suitability of stem cell based therapy for the reconstruction of lung tissue after damage. New studies have attempted to discover the mechanism involved in the repair of lung damage and create a basic science to be used in stem cell therapy for lung diseases (7).

## Materials and Methods

The present animal experiments were approved by the Institutional Ethical Committee of Islamic Azad University, Mashhad, Iran. The rabbits were sacrificed. The rabbit lung tissues were decellularized and prepared for use as a scaffold for blastema cells. Tissues were delivered to the laboratory using phosphate buffered serum (PBS) solution. Physical and chemical procedures were considered for the decellularization of this tissue.

Sodium dodecyl sulfate (SDS) (CinnaGen, Tehran, Iran) in phosphate buffered serum (PBS) (CinnaGen, Tehran, Iran) was added to the specimens for 24 hours. 1% Triton X-100 (CinnaGen, Tehran, Iran) solution in PBS was added to the mixture for 12 hours (manual mixer at room temperature). The specimen was placed in PBS for 2 hours (8). Scaffolds were placed in 70% ethanol for sterilization for 30 minutes at 37˚C. This procedure was carried out under a laminar hood (Pars Pajouhesh, Iran). Finally, scaffolds were washed with sterile distilled water; afterwards they were immersed in a sterile PBS solution for one hour.

To provide blastema tissue, the hairs on the back and front of the rabbit’s ears were removed by a hair removing cream and some holes punched using 10% lidocaine for local anesthesia. Thirty minutes after the administration of the lidocaine, some holes, 2 mm in diameter, were punched in the middle parts of the ear and away from the blood vessels. Seventytwo hours after the first punch, a second punch, 4mm in diameter, was made around the first one, so the blastema ring was cut from the ear of the animal. The samples were washed three times in plates with physiologic serums (0.9% sodium chloride).

Scaffolds were placed in a blastema ring for penetration of blastema cells into the lung scaffold. The scaffolds with the blastema ring were transferred to a 12 well-plate (Orange Scientific, Belgium) in Dulbecco’s Modified Eagle,s Medium and incubated at 37˚C in 5% CO_2_. The samples were fixed with Bouin,s fixator and then stained with hematoxilin-eosin (H&E) and hematoxilin weigert-peak indigo carmine (H&P) The samples were evaluated under a light microscope (Olympus, Japan: IX70), and Scanning Electron Microscope (SEM) (Leo-910, Germany). 4'-6-Diamidino- 2-phenylindole (DAPI) is known to form fluorescent complexes with natural doublestranded DNA, showing fluorescence specificity for AT, AU and IC clusters. Harvested cells were washed once with PBS and then re-suspended in PBS containing 0.1% Triton X (to induce holes in the cells’membranes in order to increase permeability) and incubated for 10 minutes on ice. Cells were then spun down and re-suspended at 5000 cells/µl in 4% PBS buffered paraformaldehyde solution containing 10 µg/ml DAPI (Sigma, Germany). 10 µl of this suspension was placed on a glass slide and covered with a coverslip. The morphology of the cells’ nuclei was observed using a fluorescence microscope (Olympus BH Series) at an excitation wavelength of 350 nm. The samples were assessed on the 15^th^ day after culturing.

## Results

**Fig 1:**
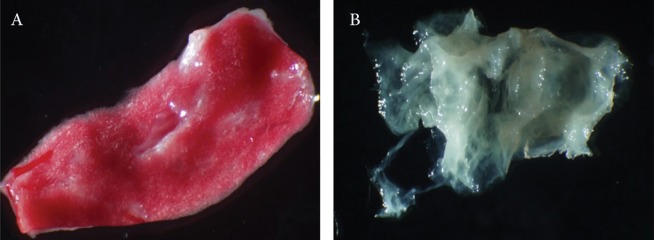
A. Appearance of rabbit lung tissue. B. Decellularized rabbit lung tissue as scaffold.

**Fig 2:**
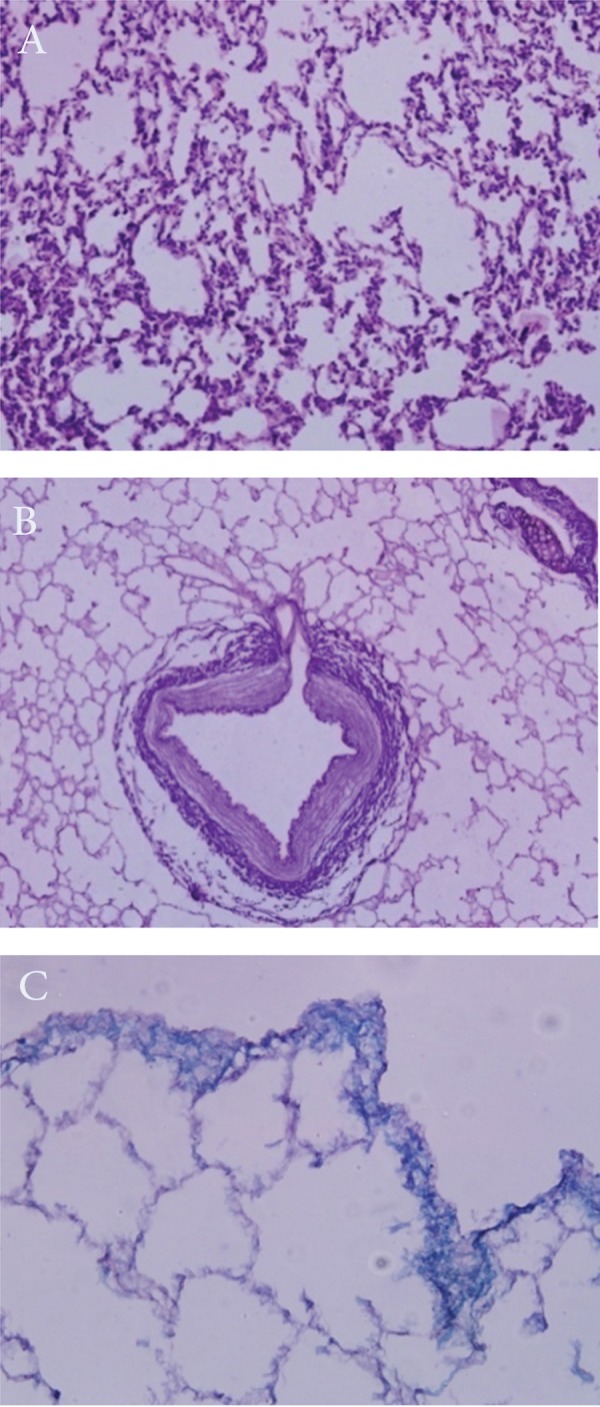
A. Before. B. After decellularization of rabbit lung tissue (A: H/E ×40 -B: H/E ×20). C. Decellularized rabbit lung tissue (H/P ×100).

**Fig 3:**
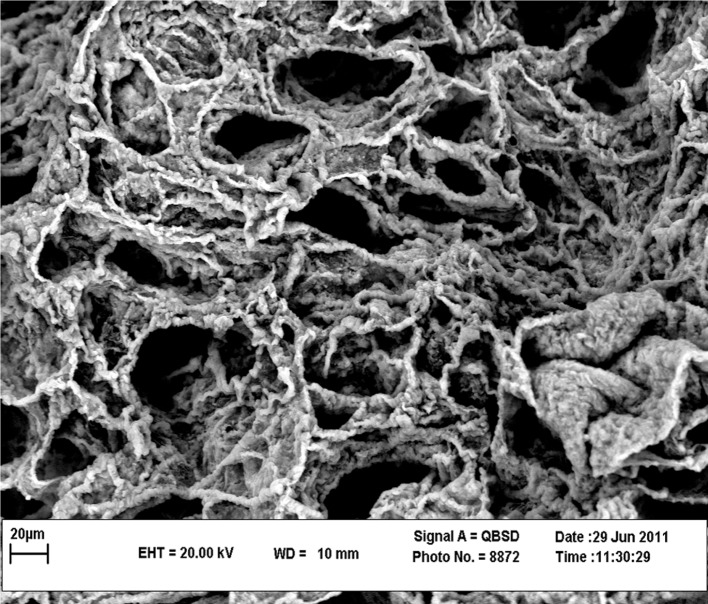
Scanning electron micrographs of the decellularized rabbit lung tissue showed that collagen and elastin fibers in the connective tissue were intact

**Fig 4:**
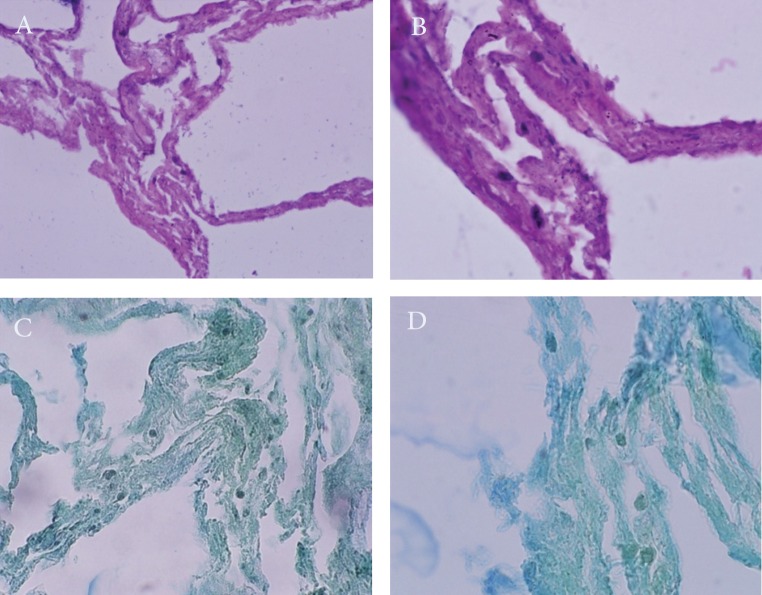
A and B. Migration and permeation of the cells from the blastema ring into the Scaffold (A: H/E ×100 -B: H/E ×400). C and D. Hematoxyline Weigert -peak indigo carmine staining on the 15^th^ day after culturing (C: H/P ×100 -D: H/P ×400).

**Fig 5:**
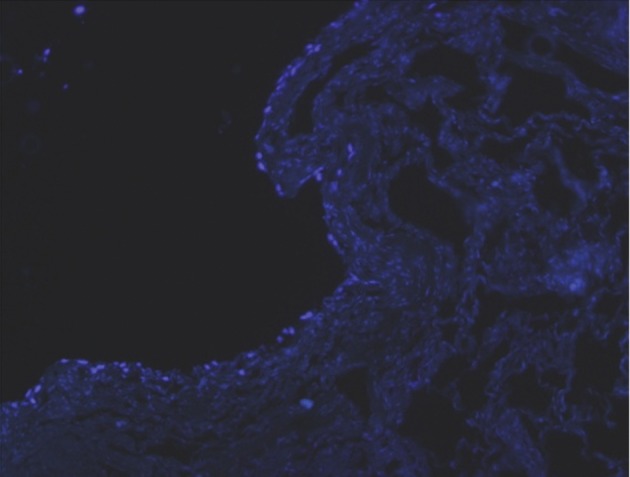
Viable cells stained with DAPI method on the 15^th^ day after culturing.

Macroscopic imaging of the rabbit lung tissue before decellularization is shown in figure 1A. The decellularization of this tissue was completely achieved using SDS 1% as noticed in figure 1B. Microscopic features of the rabbit lung tissue before and after decellularized process are shown in figure 2. Scanning electron micrographs of the decellularized rabbit lung tissues showed that the overall structure of rabbit lung tissues after decellularization (Fig 3). The microscopic sections examined on the 15^th^ day after initial seeding showed the migration and penetration of numerous of cells (Fig 4). The DAPI method showed nuclei of these cells stained blue on the 15^th^ day after culturing (Fig 5).

## Discussion

Tissue engineering is based on three basic combinations of scaffold, stem cells, and growth factors (3, 9). In human organs, cells proliferate on porous matrices that are called extracellular matrix (ECM). In a similar way, in tissue engineering methods cells proliferate in porous scaffolds for the three dimensional reconstructions of tissues (10). EMC can be used for tissue engineering in regenerative medicine. In recent years, ECM has been developed as a biological scaffold to use in engineering functions (11). The ECM materials that are used in tissue engineering are collagen, fibronectin, elastin, laminin,and lucosamineglycans (12).

Collagen can be used in tissue engineering studies as scaffold due to its high compatibility. There are many research studies about the effects of collagen on differentiation of cells inside the scaffold (13). In a study by Nillesen et al. (14) subcutaneously implanted scaffold consisting of type 1 collagen, heparin, fibroblastic growth factor (FGF) and vascular endothelial growth factor (VEGF) were placed in back of a wistar mouse. The researchers established that a combination of FGF and VEGF increased angiogenesis.

Special characteristics are needed for scaffolds: biocompatibility, a controlled rate of degradation, proper porosities, and an appropriate mechanical and chemical foundation. In tissue engineering, scaffolds should be as similar as possible to the natural environment with regards to the physiological, biochemical and biophysical conditions for cell penetration and proliferation (15, 16). Scaffolds should have open cavities that are connected together so scaffolds have increased surface/volume ratio.

These properties of scaffold lead to migration, proliferation, and differentiation of cells. Porosity of the scaffold accelerates angiogenesis hence cells interact better with surrounding tissue and drain waste materials. Capillary formation and penetration within the scaffold becomes possible by these routes. The chemical properties and topography of scaffolds control proliferation of viable cells on scaffolds (17). In this experimental study, we attempted to produce rabbit lung three-dimensional scaffold. Lung tissue was selected because of its high porosity which is an important property in tissue engineering. Lung tissue has elastin and collagens fibers (18). These fibers composed the main part of scaffold. Results from the evaluation of the scaffold structure by electron microscope showed that collagen and elastin fibers were intact. Since these fibers can have induction effects, their preservation is important. Bioscaffold of rabbit lung tissue preserved its overall structure even after 15 days from the initial seeding. Migration and permeation of blastema cells into the lung tissue scaffold was also observed. Previous studies have shown that decellularized scaffolds can provide an appropriate environment for cell penetration and proliferation similar to our study and the pluripotential capacity of stem cells for lung tissue repair has been investigated (19).

## Conclusion

Rabbit lung tissue scaffolds have a high percent of porosity. The cells migrated toward and permeated inside the scaffold.
